# Molecular Dynamics Simulation Research on Fe Atom Precipitation Behaviour of Cu-Fe Alloys during the Rapid Solidification Processes

**DOI:** 10.3390/ma17030719

**Published:** 2024-02-02

**Authors:** Xufeng Wang, Xufeng Gao, Yaxuan Jin, Zhenhao Zhang, Zhibo Lai, Hanyu Zhang, Yungang Li

**Affiliations:** College of Metallurgy and Energy, North China University of Science and Technology, Tangshan 063210, China; wangxf@ncst.edu.cn (X.W.); ruoyichen2021@163.com (X.G.); j1137025165@163.com (Y.J.); 13053334352@163.com (Z.Z.); 247537441@163.com (Z.L.); 15631867824@163.com (H.Z.)

**Keywords:** Cu-Fe alloy, Fe atom precipitation, molecular dynamics simulation

## Abstract

To explore the crystalline arrangement of the alloy and the processes involving iron (Fe) precipitation, we employed molecular dynamics simulation with a cooling rate of 2 × 10^10^ for Cu_100-X_Fe_X_ (where X represents 1%, 3%, 5%, and 10%) alloy. The results reveal that when the Fe content was 1%, Fe atoms consistently remained uniformly distributed as the temperature of the alloy decreased. Further, there was no Fe atom aggregation phenomenon. The crystal structure was identified as an FCC-based Cu crystal, and Fe atoms existed in the matrix in solid solution form. When the Fe content was 3%, Fe atoms tended to aggregate with the decreasing temperature of the alloy. Moreover, the proportion of BCC crystal structure exhibited no obvious changes, and the crystal structure remained FCC-based Cu crystal. When the Fe content was between 5% and 10%, the Fe atoms exhibited obvious aggregation with the decreasing temperature of the alloy. At the same time, the aggregation phenomenon was found to be more significant with a higher Fe content. Fe atom precipitation behaviour can be delineated into three distinct stages. The initial stage involves the gradual accumulation of Fe clusters, characterised by a progressively stable cluster size. This phenomenon arises due to the interplay between atomic attraction and the thermal motion of Fe-Fe atoms. In the second stage, small Fe clusters undergo amalgamation and growth. This growth is facilitated by non-diffusive local structural rearrangements of atoms within the alloy. The third and final stage represents a phase of equilibrium where both the size and quantity of Fe clusters remain essentially constant following the crystallisation of the alloy.

## 1. Introduction

Cu-Fe alloys are significant amorphous alloy materials. Owing to their thermal stability and mechanical properties [1,2,3], such alloys have been extensively adopted in electronics, aerospace, automobiles, and other fields. As is well known, changes in the Fe content of Cu-Fe alloys have a significant influence on the alloy structure and properties. Elevating the Fe content within the Cu-Fe lattice serves to augment both the stability of the lattice and the material’s hardness and strength. Nevertheless, this enhancement comes at the expense of reduced material toughness and plasticity, resulting in a more brittle fracture surface. Simultaneously, the wear resistance of Cu-Fe alloys demonstrates an increase proportional to the rising Fe content [4,5]. However, with the increasing Fe content, the resistivity of Cu-Fe alloys will gradually increase, causing the alloy's conductivity to decrease. This phenomenon can be attributed to the higher resistivity of Fe crystals compared to Cu crystals. Additionally, the formation of Fe cluster structures disrupts the integrity of the crystal structure, further contributing to the increased resistivity and reduced conductivity of the alloy [6,7,8]. Currently, the utilisation of rapid solidification technology for researching and producing typical immiscible alloys like Cu-Fe alloys holds great potential. The crystal structure of Cu-Fe alloys will change with increasing Fe content during rapid cooling. Cu-Fe alloys with low Fe content (Fe content is less than 3%) present a solid solution structure, which renders the formation of complex polycrystalline structures difficult. With a high Fe content (Fe content is greater than 3%), a phase separation structure is exhibited, and the Cu and Fe form separate crystal regions [9,10]. In the high-temperature crystalline state, Cu-Fe alloys exhibit a polycrystalline structure and a serialised microstructure. However, when subjected to rapid cooling, they transform into an amorphous structure with local order. The change in cooling rate can directly result in a significant alteration in the characteristics of the Fe-rich phase. During heat treatment, the morphology of the Fe-rich phase of a Cu-Fe alloy in a water-cooled solidified state will change from nanometer-sized spheres to micron-sized multi-branch petal shapes [11,12]. Currently, most experimental studies focus on the morphology and mechanical properties of Cu-Fe alloys, but there is limited research on the precipitation mechanism of Fe clusters. The precipitation of Fe clusters is crucial for the performance of alloys. Molecular dynamics simulation is a highly valuable method for investigating the microscopic properties of crystalline materials and is extensively utilised in the examination of metal clusters and metal nanoparticles [13,14]. Starting from the atomic scale, Xu et al. conducted a thorough analysis of the effects of various contents and cooling rates on solidification nucleation during rapid solidification. They further gained insight into the nucleation mechanism at the atomic scale [15]. J. Phys et al. investigated the structural evolution mechanism of Cu clusters and Fe clusters during the solidification process of a Cu-Fe alloy using molecular dynamics simulation. The nanoclusters were characterised using adaptive adjacency relationships, radial density distribution, and potential energy. They conducted comprehensive research on the structural units and bond energy of Cu-Fe alloys and presented their evolution process and nanoparticle morphology. The research results showed that Fe and Cu are immiscible. Solidification is closely related to solid volume and other factors [16]. However, the influence of the Fe doping concentration in the Cu matrix has not been thoroughly studied. Currently, there is a substantial body of literature examining the growth mechanism of Fe-rich phases and clusters, as well as their influence on alloys. However, there are few articles that systematically describe the formation process of Fe clusters and the impact of changes in Fe content on Fe cluster formation in low-Fe environments. Using molecular dynamics simulation, an investigation was conducted into the distribution and morphological changes of Fe atoms in Cu-Fe alloys with different Fe contents (1%, 3%, 5%, and 10%) under rapid cooling (2 × 10^10^ K/s). With the analysis of the structural changes of Cu-Fe alloys during rapid cooling, the formation process and precipitation mechanism of Fe atomic clusters with different Fe contents were investigated. Additionally, the structural changes of the alloys were analysed through the relationships between statistical average potential energy, radial distribution function, coordination number, and mean square displacement, as well as the changes in the alloy temperature. At the same time, by means of the atomic structure visualisation technology, the size and number changes of Fe clusters were statistically and analytically analysed, so as to explore the influence mechanism of Fe atom precipitation during the rapid cooling process of Cu-Fe alloys with different Fe contents. Investigating the distribution and segregation mechanisms of Fe atoms within alloys holds significant importance. It provides essential guidance for optimising the manufacturing process and enhancing the performance of Cu-Fe alloys.

## 2. Simulation Method

Based on existing research, the Cu_100-X_Fe_X_ (where X represents 1%, 3%, 5%, and 10%) alloy model was established using Atomsk (Pierre Hirel 2010—Version 0.11) (The Swiss-army knife of atomic simulations) software [17]. Specifically, the unit cell of the Cu single crystal was constructed first and then copied to generate a 30 × 30 × 12 supercell. There were 43,200 Cu atoms in total in the simulation box, of which 432, 1296, 2160, and 4320 were replaced randomly by Fe atoms to create the alloy model needed for simulation. Subsequently, MD simulation analysis was conducted using the large-scale atomic/molecular massively parallel simulator (LAMMPS) designed and developed by Sandia National Laboratory, Albuquerque, NM, USA [18]. During the simulation process, the separation of Fe atoms from Cu-Fe alloys was modelled using the interaction potential function introduced by Bonny et al. [19], which has been commonly applied to investigate the phase separation phenomenon in Fe-Cu alloys over the past several years. The NPT ensemble was implemented in all MD simulation steps, and a time step of 2 fs was selected. The system pressure was fixed at 0 bar, and the Nose-Hoover algorithm was employed to regulate both the pressure and temperature of the system. The simulation was conducted under periodic boundary conditions. The model first ran 300,000 time steps at 2200 K so as to allow for the atoms to diffuse fully and the alloy system to reach the equilibrium state. The alloy was then cooled to 300 K at a rate of 2 × 10^10^ K/s. The rapid cooling process was achieved through the specification of a total number of time steps for the simulation, which was set to 4.75 × 10^7^ in this study. The structural information and kinetic and thermodynamic parameters of the alloy system were recorded in the simulated cooling process. Finally, OVITO (version 3.8.3.) [20] software was used to visualise the simulation results, and common neighbour analysis and cluster analysis were performed to examine the crystal microstructure, formation, and growth patterns of Fe clusters in the simulation process.

## 3. Results and Discussion

### 3.1. The Influence of the Fe Content on the Cu_95_Fe_5_ Alloy Structure

#### 3.1.1. Average Atomic Potential Energy

**Figure 1:**
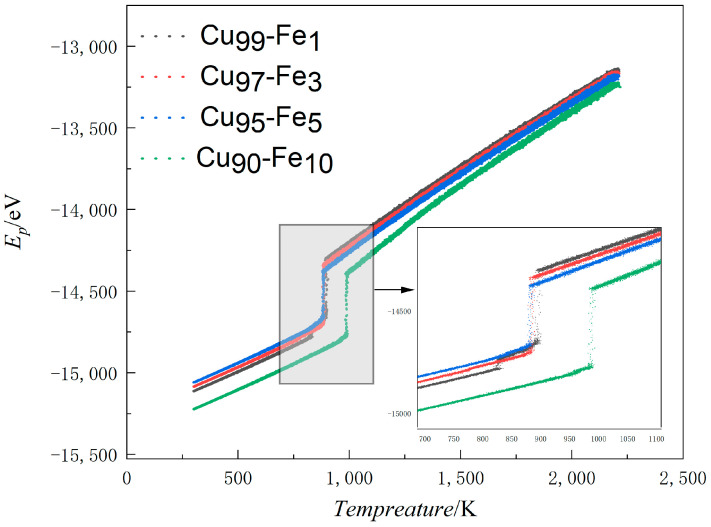
Average atomic potential energy-temperature variation line (APE-Temp variation line) for Cu-Fe alloys.

Curves depicting the variation in APE (average atomic potential energy) with temperature serve as an accurate representation of microstructural changes that occur during rapid cooling. These curves offer a clear and straightforward means of analysing the phase transition process within the system [21,22]. Generally, if a curve veers away from a straight line without any sudden shifts, it suggests that the system is amorphous. However, if the curve veers away from a straight line and there are sudden shifts, it suggests that the system is crystalline [23,24]. The plot in Figure 1 shows the average potential energy curves for Fe contents of 1%, 3%, 5%, and 10% as a function of temperature.

During the simulation process, as the system’s temperature decreases, the intensity of the thermal motion of atoms in the system also decreases. The gradual decrease in speed facilitates the combination of atoms, leading them to combine with each other, thus forming ordered structures. The formation of this ordered structure results in a decrease in system volume. When the volume of the system decreases to a certain point, the local cluster structure formed in the alloy will rapidly disintegrate in order to minimise the energy of the system. The atoms will then recombine into a more stable structure, leading to crystallization. This process is accompanied by a sudden decrease in the system’s energy. Manifested as a sudden change in the average atomic potential temperature curve. At Fe content levels of 1%, 3%, 5%, and 10%, the APE-Temp change lines exhibited temperatures of 894 K, 887 K, 887 K, and 994 K, respectively, indicating the formation of crystals in the system. The APE-Temp variation line suggests that as the Fe content in the alloy increased, the average atomic potential energy decreased, resulting in a more stable system structure.

#### 3.1.2. Analysis of Crystal Structure

**Figure 2:**
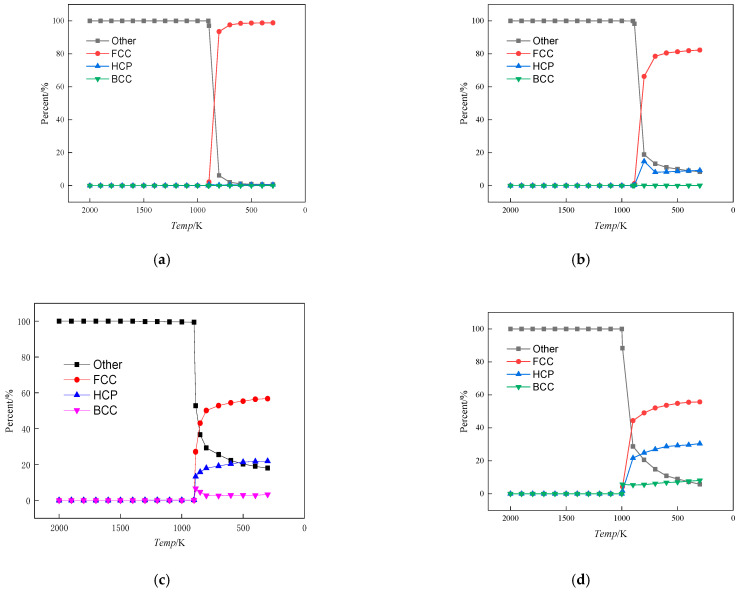
Crystal type ratio versus alloy temperature change curve. (**a**) Cu_99_Fe_1_, (**b**) Cu_97_Fe_3_, (**c**) Cu_95_Fe_5_, (**d**) Cu_90_Fe_10_.

To further investigate the effect of Fe content on the alloy structure during solidification, the crystal configuration was analysed as shown in Figure 2. The various crystal structures in the system are determined using common neighbour analysis (CNA) in OVITO software (version 3.8.3). The CNA is an algorithm to compute a fingerprint for pairs of atoms that is designed to characterise the local structural environment. Typically, the CNA is used as an effective filtering method to classify atoms in crystalline systems, with the goal of getting a precise understanding of which atoms are associated with which phases and which are associated with defects. In this paper, the modifier selected is adaptive CNA (with variable cutoff), which determines the optimal cutoff radius automatically for each individual particle.

As shown, when the system temperature was in the interval from 2000 K to the phase transition temperature (the phase transition temperatures of Cu_99_Fe_1_, Cu_97_Fe_3_, Cu_95_Fe_5_, and Cu_90_Fe_10_ were 894 K, 887 K, 887 K, and 994 K, respectively), the system existed in a liquid state, devoid of any crystalline structures. The Other category represents atoms that do not belong to any specific crystalline structure and are generally considered defects, occupying positions outside the lattice sites. At the phase transition temperature, there was a rapid decline in the proportion of other type atoms, concurrent with a substantial increase in the proportion of the FCC crystal structure. Figure 2 shows that at a temperature of 300 K within the alloy, the proportion of the FCC crystal structure diminished as the Fe content in the alloy increased. The crystal structure of Cu in this system was FCC, and the proportion of FCC crystal structure decreased, indicating a decrease in the percentage of Cu crystals or a decline in the proportion of Cu solid solution. Similarly, the crystal structure of Fe in this system was BCC, and there was almost no BCC crystal in the alloy when the Fe content was between 1% and 3%, indicating that Fe atoms may not have been clustered or clusters of smaller Fe atoms were involved in the formation of HCP and FCC crystal structures. The observation of the BCC crystal structure at 5% Fe content, as depicted in the temperature change curve, reveals that this structure initially increased and then decreased with the phase transition temperature. This phenomenon suggests that clusters formed by Fe atoms exhibited an initial increase followed by a decrease. Considering the subsequent process of Fe cluster formation, it can be concluded that the abrupt decrease in the BCC crystal structure at the onset of crystallisation was due to the continuous formation of Fe clusters within the alloy before crystallisation occurred. Before the alloy crystallised, it contained both large and small Fe clusters within the system. As the alloy reached the crystallisation temperature, some of the smaller clusters decomposed into individual atoms or condensed as a whole. These condensed clusters adopted the FCC and HCP crystal structures, with Cu atoms serving as the basis for atom arrangement to achieve greater compactness. Ultimately, this process led to the formation of the Cu matrix with embedded Fe atoms, primarily in the FCC and HCP crystal types. The BCC crystal structure of the alloy with 10% Fe content increased gradually as it cooled down from the phase transition temperature, unlike the alloy with 5% Fe content. This slow increase was caused by a few iron (Fe) atoms near the iron clusters combining to form larger iron clusters.

#### 3.1.3. Radial Distribution Function Analysis

**Figure 3:**
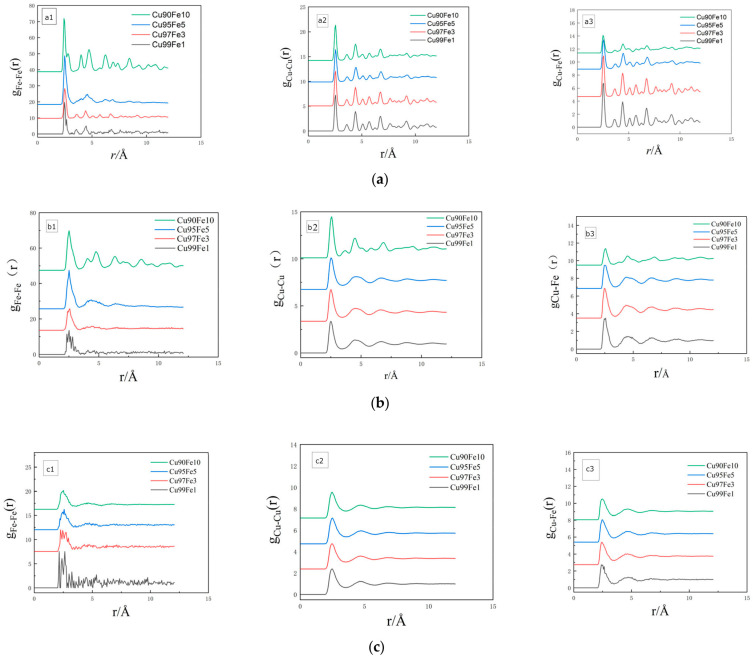
Plot of radial distribution function of alloy at different temperatures. (**a**) 300 K, (**b**) crystallisation temperature, (**c**) 2000 K.

The radial distribution function, expressed as g(r), is a statistical parameter reflecting the distribution characteristics of system atoms and $g_{\alpha -\beta }(r)$ represents the ratio of the probability of finding an atom β at a fixed atomic distance α from radius r to the conditional probability [25,26,27]. The radial distribution function serves as a valuable tool for distinguishing between liquid, crystalline, and amorphous states in a material. It offers structural insights, including atomic radius, inter-atomic spacing, and coordination number. Through the analysis of peaks in the radial distribution function curve, the strength of inter-atomic interactions can be assessed, and the extent of short- and mid-range ordering in the material’s atomic arrangement can be determined [28,29]. The radial distribution functions of Cu_99_Fe_1_, Cu_97_Fe_3_, Cu_95_Fe_5_, and Cu_90_Fe_10_ alloys were calculated at 2000 K–300 K with a cooling rate of 2 × 10^10^ K/s, and the results are shown in Figure 3.

The results of the radial distribution function analysis, g(r), conducted in the present study on Cu-Fe alloy systems with varying Fe contents, cooled at a rate of 2 × 10^10^, reveal specific temperature intervals. When the Fe content was 1%, 3%, 5%, and 10%, corresponding to temperature ranges of 2000 K–894 K, 2000 K–887 K, 2000 K–887 K, and 2000 K–994 K, respectively, the first peak in the radial distribution function exhibited a distinct non-spiky pattern with clearly resolved symmetric peaks. These characteristics are indicative of the system being in a liquid state within these temperature intervals [30]. Upon further analysis, it was evident that this conclusion aligns closely with other physical properties and demonstrates high experimental reliability. In the subsequent temperature intervals, the shape of the first peak in the radial distribution function for systems with varying Fe contents all displayed symmetric, sharp peaks. These peaks were notably more pronounced than the other peaks, which strongly suggests that the system transitioned into a crystalline state [31,32]. In light of the aforementioned analyses, the phase transition temperature for each alloy system could be reliably derived.

Upon analysing the radial distribution function g(r), the first peak values of $g_{Fe-Fe}\left(r\right)$ were found to be higher than those of $g_{Cu-Cu}(r)$ and $g_{Cu-Fe}(r)$ for alloys with varying Fe contents and temperatures. As the temperature of the alloy decreased, the difference between the Fe-Fe atomic pairs of the first peak and the Cu-Fe and Cu-Cu atomic pairs of the first peak became increasingly larger, and the Cu-Cu atomic pairs of the first peak gradually exceeded the Cu-Fe atomic pairs of the first peak. At an alloy temperature of 300 K, it was observed that the higher the Fe content in the alloy, the greater the first peak value for Fe-Fe atomic pairs in the radial distribution function. These findings indicate a substantial attraction between Fe-Fe atom pairs, in contrast to the relatively weaker attraction between Cu-Fe and Cu-Cu atom pairs. The increase in the number of Fe atoms within the alloy plays a significant role in facilitating the formation of Fe clusters. The analysis of the value of the second peak of the radial distribution function provided information about the changes in the structure and properties of the system with temperature [33]. In the Cu_95_Fe_5_ and Cu_90_Fe_10_ alloy systems, it is clear that as the temperature decreased, the value of the second peak of the $g_{Fe-Fe}(r)$ curve increased. After the phase transition temperature, the values of the $g_{Fe-Fe}(r)$ curve were consistently above 1, indicating that most of the first and second nearest neighbours of Fe atoms were occupied by Fe atoms at this stage. The second peaks of $\;g_{Cu-Fe}(r)$ and $g_{Cu-Cu}(r)$ decreased with the decrease in temperature until dropping below 1 at 300 K, indicating that at this time, the probability of Cu atoms and Fe atoms appearing in the second nearest-neighbour layer of Cu atoms was the same as the conditional probability, and the interatomic correlation was low. In summary, the following conclusions can be drawn from the analysis: The interaction force between Fe-Fe atoms was notably stronger than that between other atom pairs; the alloy’s Fe-aggregation tendency increased with the increase in the number of Fe atoms; and the aggregation of Fe atoms was an active behaviour. When the temperature of the alloy decreased, the probability of the appearance of Fe atoms around the Fe atoms increased gradually, which could be attributed to the fact that a closer interaction could be established between Fe atoms with equal charges. In this process, the proportion of the first and second nearest neighbours of Fe atoms occupied by Fe atoms also increased, thereby promoting the formation of Fe atoms into clusters.

#### 3.1.4. Allotropic Analysis

**Figure 4:**
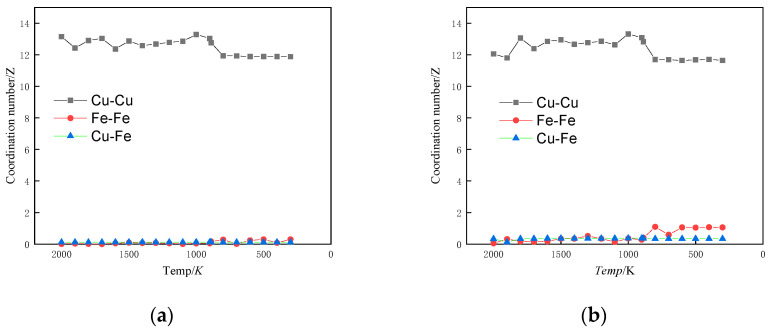

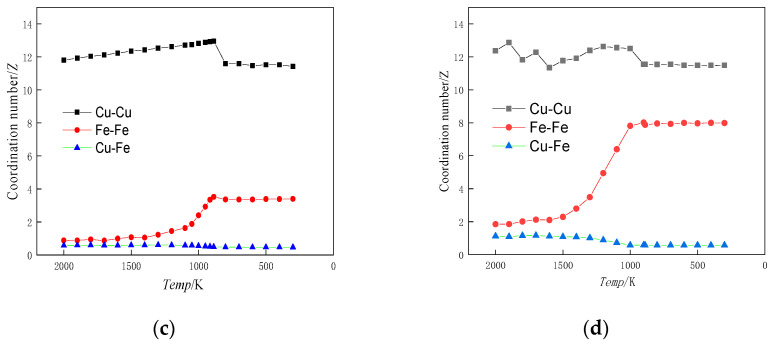
Coordination number versus temperature profile. (**a**) Cu_99_Fe_1_, (**b**) Cu_97_Fe_3_, (**c**) Cu_95_Fe_5_, (**d**) Cu_90_Fe_10_.

The coordination number (CN) indicates how many molecules are found in the range of each coordination sphere. Integrating g(r) in spherical coordinates to the first minimum of the RDF will give the coordination number of a molecule. The coordination number is a parameter that provides insight into the local atomic bonding within alloys. It is indicative of the short-range ordering of atomic arrangements and describes the proximity or closeness of atoms to a central atom. The coordination number is a statistical method for exploring the arrangement pattern of atoms’ nearest neighbours, and an increase in the coordination number indicates an increase in the local packing density of atoms [34,35]. Figure 4 shows the variation of atomic coordination number with temperature under simulated conditions.

When the alloy contained 1% Fe, the coordination numbers of Fe-Fe and Cu-Fe atoms remained relatively constant as the temperature decreased. However, once the temperature reached the phase change point, the coordination number of Cu-Cu atoms suddenly decreased. This indicates that the atomic arrangement became denser and the alloy underwent crystallisation. At this point, the crystal structure of Cu dominated the alloy, and Fe clusters did not form. When the alloy contained 3% Fe and reached the phase transition temperature, the coordination number of Fe-Fe atoms increased. This signifies that Fe-Fe atoms tend to aggregate at this specific temperature and composition. However, the coordination number of Cu-Fe atoms notably remained largely unchanged during this phase transition. Given the phenomenon that the proportion of BCC crystal structure was basically unchanged with the decrease in temperature in Figure 2b, an observation can be made that when the Fe content was 3%, although the Fe-Fe atoms had the tendency to aggregate with each other, the Cu atoms accounted for a larger proportion of the Fe atoms. Moreover, the clusters formed by Fe atom aggregation were solidly dissolved in the Cu matrix of the FCC crystal structure, and the structure of the alloy was still dominated by the Cu crystal structure, which is the reason for the basically unchanged coordination number of the Cu-Fe atoms. This is also the reason why the Cu-Fe atomic coordination number was basically unchanged [36]. When the Fe content was between 5% and 10%, the trends of Fe-Fe, Cu-Fe, and Cu-Cu atomic coordination numbers with temperature change were essentially the same, and the change was more obvious for the Fe content of 10% than that of 5%. When the temperature of the alloy reached the phase transition temperature, the coordination number of Cu-Cu atom pairs suddenly decreased, and crystals were formed. Before reaching the phase transition temperature, the coordination number of Fe-Fe atoms steadily increased as the temperature decreased. In contrast, the coordination number of Cu-Fe atoms decreased gradually before the phase transition temperature. These trends suggest that in the liquid state, Cu-Fe atoms were dispersed, while Fe-Fe atoms began to exhibit increasing attraction and aggregation. This process continued until the phase transition temperature was reached. After the phase transition temperature, the coordination numbers of these atoms remained relatively stable, indicating a distinct change in the state of the alloy.

#### 3.1.5. Mean-Square Displacement Analysis

Atomic diffusion is the process by which atoms move from one of their positions to another across lattice vacancies or other defective sites during thermal movement within a crystal [37]. The atomic diffusion coefficient is a physical quantity that describes the rate of atomic diffusion motion [38]. In molecular dynamics simulations of alloy systems, one common method to calculate the diffusion coefficients of atoms is by monitoring the mean square displacements (MSD) of atoms throughout the simulation [39].

(1) $$D^{*}=\frac{<r^{2}(t)>}{2Nt}$$

where N—the dimension of the simulation system, N = 3 for this system; t—the simulation time, ps; *r*(t), *r*(0)—the position of the atom at time t and the initial position of the atom, respectively.

(2) $$MSD=<r^{2}(t)>=\frac{1}{N}\sum_{i}^{N}<{\left|r_{i}\left(t\right)-r_{i}(0)\right|}^{2}>$$

where MSD is the mean square displacement;

Combining Formulas (1) and (2), the following can be obtained:

(3) $$D^{*}=\frac{MSD}{6t}\;\;\;\to \;\;MSD=6D^{*}t$$

The diffusion coefficient of Fe atoms was 1/6 of the slope of the relationship curve between MSD and time t.

**Figure 5:**
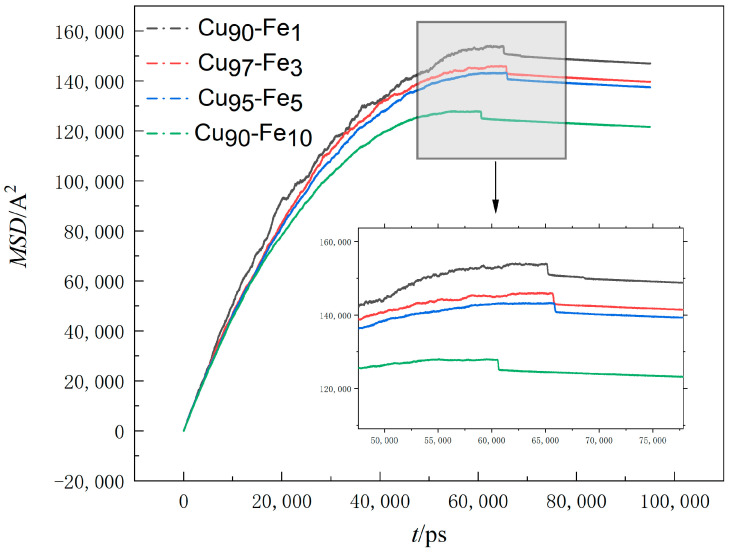
The variation of MSD with time.

Figure 5 summarises the MSD values of Fe atoms along the X, Y, and Z directions at a cooling rate of 2 × 10^10^ K/s.

The observed decrease in the slope of the MSD (Mean Square Displacement) versus the time curve as time progressed suggests that the diffusion rate of Fe atoms within the system decreased over time [40]. This phenomenon can be attributed to the diminishing temperature of the system as time elapses. The significant subcooling effect caused a reduction in the thermal mobility of the atoms, leading to a decrease in the range of atomic movement. Additionally, from the visual observations, it became apparent that at any given moment, a higher Fe content within the alloy corresponded to a smaller mean square displacement and a reduced slope in the MSD vs. time curve. This further signifies that the diffusion rate of Fe atoms decreased as Fe content increased, and the range of atomic movement became more limited. At 55,000 ps–60,000 ps, the slope of the curve was close to 0, indicating that the diffusion rate of Fe atoms at this stage gradually decreased to 0. However, when analysed in context, non-diffusive local atomic structural rearrangements dominated the structural changes within the system during this period, making this phase an important stage in the formation of large Fe clusters. Here, non-diffusive atomic structure rearrangement refers to the fact that although an atom moves, it is in the bondage of the surrounding atoms; that is, the local structure of the atom and its surroundings is not changed. The aggregation and condensation of small clusters is a specific manifestation of this. At the times of 65,822 ps, 65,762 ps, 65,936 ps, and 60,682 ps, respectively, the curve appeared to be broken, at which time crystallisation took place, followed by minimal changes in the trace, which was mainly due to the low temperature of the system, with the cooling rate being too fast, resulting in a small range of atomic motion.

### 3.2. Formation Mechanism of Fe Clusters in Cu-Fe Alloys during Rapid Cooling

**Figure 6:**
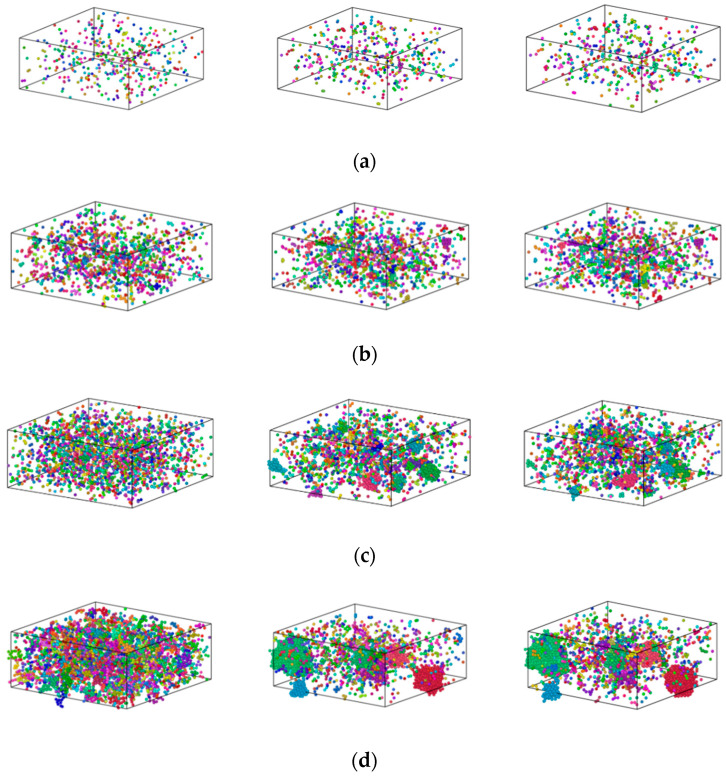
Distribution of Fe atoms in the Cu_100-X_Fe_X_ alloy (**a**) Cu_99_Fe_1_, (**b**) Cu_97_Fe_3_, (**c**) Cu_95_Fe_5_, and (**d**) Cu_90_Fe_10_. Different colors represent clusters of different sizes.

Figure 6 shows plots of the distribution of Fe atoms for Cu_99_Fe_1_, Cu_97_Fe_3_, Cu_95_Fe_5_, and Cu_90_Fe_10_ alloys at three distinct temperature points: 2000 K, the phase transition temperature, and 300 K, respectively. The square in Figure 6 represents the simulation box with dimensions of 108.42 × 108.42 × 43.368 Å^3^.

The clusters in the system were determined using cluster analysis in OVITO software (version 3.8.3.). This modifier decomposes the particles into disconnected groups (so-called clusters) based on the selected neighbouring criterion. The neighbouring criterion can be distance-based (cutoff range) or topology-based (bond network); this paper selected distance-based (cutoff range). The cutoff range is determined by selecting the first minimum value of RDF. A cluster is defined as a set of connected particles, each of which is within the (indirect) reach of the other particles in the same cluster. Thus, any two particles from the same cluster are connected by a continuous path consisting of steps that fulfil the selected neighbouring criterion. Coloring particles by cluster gives each identified cluster a unique random colour and colours the particles according to the clusters they belong to. It helps to quickly visualise the results of the clustering algorithm.

**Figure 7:**
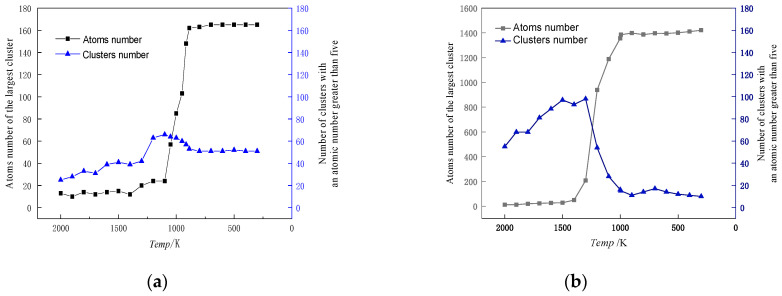
Cu_95_Fe_5_ (**a**), Cu_90_Fe_10_ (**b**) alloys at 300 K, the maximum cluster contains the number of atoms, the number of atoms greater than 5 total number of clusters versus the temperature change of the graph.

Cu_99_Fe_1_ alloy did not show the Fe atom aggregation phenomenon with decreasing temperature, and Fe atoms consistently maintained a uniformly dispersed state. Different from the distribution state of Fe atoms in the Cu_99_Fe_1_ alloy, the Fe atom aggregation phenomenon was found in the Cu_97_Fe_3_ alloy with a decrease in temperature, but the Fe clusters in the Cu_97_Fe_3_ alloy were found to be unstable after observation using visualisation software. Here, the maximum number of atoms contained in the clusters was not more than 5. Combined with the phase diagram analysis of Cu-Fe alloys, an observation can be made that when the Fe content of Cu-Fe alloys was lower than 3%, the Fe atoms mainly existed in the form of a Cu-based solid solution, which is consistent with the previous analysis and the results from prior research. When the temperature of Cu_95_Fe_5_ and Cu_90_Fe_10_ alloys was 2000 K, the Fe atoms were uniformly distributed in the system and did not show any structural non-uniformity. With the temperature reduction, the Fe atoms tended to aggregate with one another. Particularly within the temperature interval around 2000 K, which corresponded to the phase transition temperature, a gradual process occurred where Fe atoms transitioned from being dispersed as single atoms to forming small clusters. These small clusters subsequently grew in size, leading to the development of larger clusters with a non-uniform distribution. At the phase transition temperature in K, there was a temperature interval where the size of Fe clusters remained relatively stable, indicating a relatively stable system structure. To facilitate the study of cluster formation and growth, the analysis focused on Cu_95_Fe_5_ and Cu_90_Fe_10_ alloys at a temperature of 300 K when the largest clusters were present. The analysis considered the number of atoms within the largest clusters at different moments, including clusters with more than 5 atoms. This data were used to create a graph that depicts the total number of clusters and their sizes as a function of temperature change. Notably, the term “clusters” here refers to groups of atoms, and the largest cluster consistently remained within the temperature range from the phase transition temperature to 300 K, as illustrated in Figure 7.

**Figure 8:**
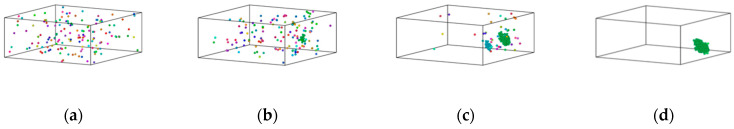
Distribution of Fe atoms in Cu_95_Fe_5_ maximal clusters at different temperatures. (**a**) 2000 K, (**b**) 1050 K, (**c**) 940 K, and (**d**) 300 K.

**Figure 9:**
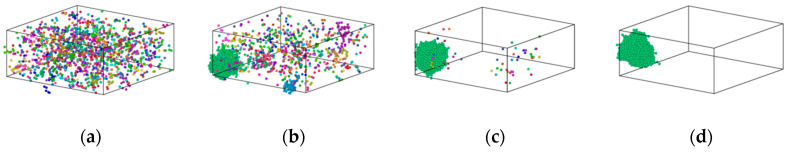
Distribution of Fe atoms in Cu_90_Fe_10_ maximal clusters at different temperatures. (**a**) 2000 K, (**b**) 1400 K, (**c**) 1000 K, and (**d**) 300 K.

In order to facilitate the investigation into the formation of clusters, the analysis focused on the growth process at a temperature of 300 K when the largest clusters were present, as depicted in Figure 8 and Figure 9. The square in Figure 8 and Figure 9 represents the simulation box with dimensions of 108.42 × 108.42 × 43.368 Å^3^.

The formation process of Fe clusters in Cu_95_Fe_5_ and Cu_90_Fe_10_ was basically the same, and the largest cluster grew rapidly in a certain temperature range, while the number of clusters decreased rapidly. The precipitation behaviour of Fe atoms could be divided into three phases. In the first phase, occurring between 2000 K and 1050 K for 5% Fe content or between 2000 K and 1400 K for 10% Fe content, the number of atoms within the largest clusters exhibited slight changes with decreasing temperature, while the total count of clusters containing more than 5 atoms significantly increased as the temperature dropped. This behaviour is a result of the interplay between the attractive forces among Fe atoms and the thermal motion of atoms. The mutual attraction of Fe atoms led to their aggregation, but at higher temperatures, intense thermal motion and a higher diffusion rate prevailed, causing an increase in the number of small clusters. The second stage, spanning from 1050 K (for 5% Fe content) to the phase transition temperature or 1400 K (for 10% Fe content) to the phase transition temperature, could be characterised by a rapid increase in the size of the largest clusters. Concurrently, the total number of clusters decreased during this stage. This observation suggests that the swift growth of clusters in this phase primarily arose from the condensation of smaller clusters. This insight was derived from the visualisation of the distribution of Fe atoms using software. For the third stage, as the alloy entered the crystallisation zone and the temperature continued to drop toward −300 °F, the increased cooling rate reduced atomic diffusion rates. As such, this inhibited the substantial growth of larger clusters due to the limited time for atomic diffusion. In summary, the persistent nucleation of Fe clusters throughout the cooling process was driven by Fe-Fe interatomic forces. The size and nucleation rate of iron clusters were influenced by atomic diffusion. Therefore, the formation of clusters at different stages was found to be a result of the combined effects of Fe interatomic forces and atomic diffusion.

## 4. Conclusions

(1) The Fe-Fe interatomic interaction force was found to be crucial for driving Fe cluster formation. The radial distribution function analysis and coordination number of the alloy during solidification demonstrate that the interaction force between Fe-Fe atoms was considerably stronger than that between other atom pairs. Further, the tendency for Fe atom aggregation increased with a higher content of Fe atoms in the alloy. The mutual attraction and aggregation of Fe-Fe is a continuous process that commences when the alloy is in a liquid state.
(2) When the Fe content reached 1%, lowering the alloy temperature did not lead to the aggregation of Fe atoms. Instead, Fe atoms maintained a uniform distribution throughout the matrix, forming a solid solution. The crystal structure remained dominated by FCC-based Cu crystals. At 3% Fe content, a decrease in alloy temperature resulted in Fe atom aggregation. However, the crystal structure, primarily based on BCC, did not undergo significant changes and continued to be dominated by FCC-based Cu crystals. When the Fe content was between 5% and 10%, the Fe atoms formed clusters as the temperature of the alloy decreased.
(3) The higher the iron content, the more apparent the clusters became, and precipitation of Fe atoms occurred in three stages. In the first stage, the increase in the number of iron clusters occurred as a result of the interplay between iron-iron atomic attraction and the thermal motion of atoms. During this phase, the size of the clusters stabilised. In the second stage, non-diffusible iron atoms underwent rearrangement influenced by the local atomic structure. This stage encompassed non-diffusive rearrangement of atoms, particularly those within condensed and growing small clusters. The third stage could be characterised by the basic stability of cluster size and number following the crystallisation of the alloy.

## Data Availability

The original contributions presented in the study are included in the article, further inquiries can be directed to the corresponding author.
